# Supplementary material for: Red blood cell indices versus serum ferritin as surrogate markers of iron deficiency during pregnancy
Source: PLoS One. 2025 Oct 29;20(10):e0334098. doi: 10.1371/journal.pone.0334098 (PMC12571326; doi:10.1371/journal.pone.0334098)
Supplement: Attachment — Submitted filename: PLOS ONE - RESPONSE TO EDITORIAL COMMENTS.pdf [file pone.0334098.s002.pdf]

PONE-D-25-15616

Red blood cell indices versus serum ferritin as surrogate markers of iron deficiency during pregnancy

Dear Editor,

Thank you for reaching out. We have addressed the queries raised as follows:

Comment

1. During our evaluation of the documents provided, we noted that your ethics approval letter did not cover the entire date range for participant recruitment and data collection. Before we can proceed further with the submission, please provide the ethics approval extension document(s) for the study. Please provide an English translation if the document is in another language.

Response

Thank you for drawing our attention to this. We have uploaded additional ethical approval documents that were obtained for proposal amendments and extensions.

Comment

2. Please report in the Methods section the site-specific recruitment period for this study in DD/MM/YYYY format.

Response

Thank you. We have reported the site-specific recruitment period in the format DD/MM/YYYY as follows: "*Study setting* – The clinical trial enrolled participants **from 10/08/2021 to 15/12/2022** from eleven public healthcare facilities in Lagos and Kano states of Nigeria" (Page 6, second paragraph).

Best regards,

Dr Ochuwa Adiketu Babah
Corresponding Author.
