# Supplementary material for: Red blood cell indices versus serum ferritin as surrogate markers of iron deficiency during pregnancy
Source: PLoS One. 2025 Oct 29;20(10):e0334098. doi: 10.1371/journal.pone.0334098 (PMC12571326; doi:10.1371/journal.pone.0334098)
Supplement: Attachment — Submitted filename: Response to reviewers.docx [file pone.0334098.s003.docx]

**PONE-D-25-15616**

**Red blood cell indices versus serum ferritin as surrogate markers of iron deficiency during pregnancy**

**PLOS ONE**

Dear Dr Oluyinka Ajibola Iyiola,
Academic Editor,
PLOS One.

Thank you for the valuable time you and the reviewers have spent to critically appraise this manuscript. We have addressed the review comments and provided a summary as a point-by-point response below. Page numbers cited are of the manuscript with tracked changes.

Thank you.

Best regards,

Ochuwa A. Babah

Corresponding Author

**Journal Requirements**

When submitting your revision, we need you to address these additional requirements.

1. Please ensure that your manuscript meets PLOS ONE's style requirements, including those for file naming. The PLOS ONE style templates can be found at https://journals.plos.org/plosone/s/file?id=wjVg/PLOSOne_formatting_sample_main_body.pdf and https://journals.plos.org/plosone/s/file?id=ba62/PLOSOne_formatting_sample_title_authors_affiliations.pdf

**Response**

Thank you. The manuscript title page has been formatted according to journal requirements by using Asterix to indicate the corresponding author and by providing only email address for the corresponding author. Page 1.

2. When completing the data availability statement of the submission form, you indicated that you will make your data available on acceptance. We strongly recommend all authors decide on a data sharing plan before acceptance, as the process can be lengthy and hold up publication timelines. Please note that, though access restrictions are acceptable now, your entire data will need to be made freely accessible if your manuscript is accepted for publication. This policy applies to all data except where public deposition would breach compliance with the protocol approved by your research ethics board. If you are unable to adhere to our open data policy, please kindly revise your statement to explain your reasoning and we will seek the editor's input on an exemption. Please be assured that, once you have provided your new statement, the assessment of your exemption will not hold up the peer review process.

**Response**

Thank you. We have rephrased our data sharing statement as follows:
The dataset analysed for this study can be accessed on Open Science Framework at: <https://osf.io/enx7z>
This statement is now in the manuscript on Page 19.

3. Thank you for stating the following financial disclosure:
Bill & Melinda Gates Foundation (Investment ID INV-017271) Grant for IVON TRIAL, the research in which this study was nested.
Please state what role the funders took in the study. If the funders had no role, please state: "The funders had no role in study design, data collection and analysis, decision to publish, or preparation of the manuscript."
If this statement is not correct you must amend it as needed.
Please include this amended Role of Funder statement in your cover letter; we will change the online submission form on your behalf.

**Response**

Done. Now on Page 19 of the manuscript.

4. Please amend your list of authors on the manuscript to ensure that each author is linked to an affiliation. Authors’ affiliations should reflect the institution where the work was done (if authors moved subsequently, you can also list the new affiliation stating “current affiliation:….” as necessary).

**Response**

Thank you. We have cross checked the manuscript and hereby confirm that this is in order.

5. If the reviewer comments include a recommendation to cite specific previously published works, please review and evaluate these publications to determine whether they are relevant and should be cited. There is no requirement to cite these works unless the editor has indicated otherwise.

**Response**

Thank you. This is not applicable as none of the reviewers recommended any specific citation.

6. Please review your reference list to ensure that it is complete and correct. If you have cited papers that have been retracted, please include the rationale for doing so in the manuscript text, or remove these references and replace them with relevant current references. Any changes to the reference list should be mentioned in the rebuttal letter that accompanies your revised manuscript. If you need to cite a retracted article, indicate the article’s retracted status in the References list and also include a citation and full reference for the retraction notice.
[Note: HTML markup is below. Please do not edit.]

**Response**

Thank you. We have checked and confirm our references to be in order.

**Reviewer #1**

A paragraph incorporating the role of ferritin and red cell indices in Fe metabolism should be added to elucidate the importance of these parameters. An explanation for the main observation that the red cell indices cannot replace ferritin estimation should also be given.

**Response**

Thank you. We have expanded the discussion section based on the comment above. We added on Page 13, last paragraph that:

Ferritin is an intracellular protein which stores in the body cells; it is thus used to assess iron stores in the body. The red blood cell indices indirectly assess iron content of the body by determining the quality of the red blood cells in terms of haemoglobin content or the size of the cells. Haemoglobin contains a Heme and a globin moiety. Heme contains iron, depicting a crucial role of iron in erythropoiesis. This study has shown indirect assessment of iron content of the body using red blood cell indices like MCH. MCHC, MCV and RDW to diagnose iron deficiency during pregnant to be inferior to the use of serum ferritin.

**Reviewer #2**

The units (g/dl) for should be g/dL in the abstract, table 1

**Response**

Thank you. We have replaced on g/dl in the manuscript with g/dL. Abstract Page 2, last paragraph; Page 10, second paragraph; Table 1 on Page 11

In the abstract conclusion, the red blood cells versus serum ferritin should be discussed.

**Response**

Thank you for the suggestion. Done. Page 3, paragraph 2 (abstract conclusion).

The unit of serum ferritin (ng/mL) should be consistent throughout and not changed to µg/mL at the discussion section.

**Response**

Thank you. The typographical error has been corrected. Page 17, Paragraph 2.

The first reference in the Reference section should be rewritten by closing up the gaps.

**Response**

This has been done. Thank you. Page 19, Reference 1.
